# Organosilica Membrane with Ionic Liquid Properties for Separation of Toluene/H_2_ Mixture

**DOI:** 10.3390/ma10080901

**Published:** 2017-08-03

**Authors:** Yuichiro Hirota, Yohei Maeda, Yusuke Yamamoto, Manabu Miyamoto, Norikazu Nishiyama

**Affiliations:** 1 Division of Chemical Engineering, Graduate School of Engineering Science, Osaka University, 1-3 Machikaneyama, Toyonaka, Osaka 560-8531, Japan; ymaeda@cheng.es.osaka-u.ac.jp (Y.M.); y.yamamoto@cheng.es.osaka-u.ac.jp (Y.Y.); nisiyama@cheng.es.osaka-u.ac.jp (N.N.); 2 Department of Chemistry and Biomolecular Science, Gifu University, 1-1 Yanagido, Gifu 501-1193, Japan; m_miya@gifu-u.ac.jp

**Keywords:** organosilica membrane, ionic liquid, hydrogen purification, organic chemical hydride, hydrocarbon separation

## Abstract

In this study, we present a new concept in chemically stabilized ionic liquid membranes: an ionic liquid organosilica (ILOS) membrane, which is an organosilica membrane with ionic liquid-like properties. A silylated ionic liquid was used as a precursor for synthesis. The permselectivity, permeation mechanism, and stability of the membrane in the H_2_/toluene binary system were then compared with a supported ionic liquid membrane. The membrane showed a superior separation factor of toluene/H_2_ (>17,000) in a binary mixture system based on a solution–diffusion mechanism with improved durability over the supported ionic liquid membrane.

## 1. Introduction

Organic chemical hydrides (OCH), which are hydrogenated aromatic hydrocarbons (e.g., methylcyclohexane), offer many advantages, such as high H_2_ content and an easy transporting system [1]. In the OCH process, the technology of H_2_ separation and purification from the ternary mixture of H_2_/aromatic hydrocarbon/cycloalkane is one of the key issues in producing pure H_2_. Intense research efforts have focused on the development of membrane separation. Various membranes, including Pd membranes [2,3,4], amorphous silica membranes [5,6], carbon membranes [7], and organosilica membranes [8,9] have been reported to show high separation performance in the OCH process. From the viewpoint of separation efficiency, hydrocarbon-selective membranes are desired in the OCH process, because concentrations of aromatic hydrocarbons and cycloalkanes are lower than that of H_2_ after the dehydrogenation reaction (e.g., C_7_H_14_ ⇄ C_7_H_8_ + 3 H_2_). However, hydrocarbon-selective membranes with high separation performance are required to produce high-purity H_2_.

In our previous study, a supported ionic liquid membrane (SILM) was prepared by an impregnation method, and subjected to the separation in the OCH process for the first time [10]. Ionic liquids (ILs) have been used in membrane separation for more than a decade due to their physical and chemical properties, such as nonvolatility, thermal stability, ability to dissolve CO_2_, and a large range of organic molecules [11,12,13,14,15]. Our SILM showed high separation factors of C_6_H_6_/H_2_ and C_6_H_12_/H_2_ in the ternary system, thus showing excellent potential as a technology for H_2_ purification in the OCH process by removing aromatic hydrocarbons and cycloalkanes simultaneously from the ternary system. However, “blow-out” of the ILs through the pores of the support membranes is an inherent problem with SILMs due to the weak capillary force for the holding of ILs in the porous support, which limits the application of SILMs. Therefore, the development of highly stable IL membranes has been a desirable goal. So far, various IL membranes, including poly-IL membranes [16,17,18,19], tough ion gel membranes [20,21,22], and chemically stabilized IL membranes [23,24] have been investigated. Among them, Vangeli et al. developed chemically stabilized IL membranes by grafting silylated ILs onto the pore surface of ceramic nanofiltration membranes, and their CO_2_/CO separation performances were evaluated [23]. A maximum separation factor of 25 was achieved at 333 K. However, it is difficult to fully fill the pores with silylated ILs by the stabilization method. Thus, their separation performances were not always equal to those of SILMs due to the presence of voids. They also concluded that further optimization was required to fill the nanofiltration pore with silylated ILs in order to improve their separation performance.

**Figure 1:**
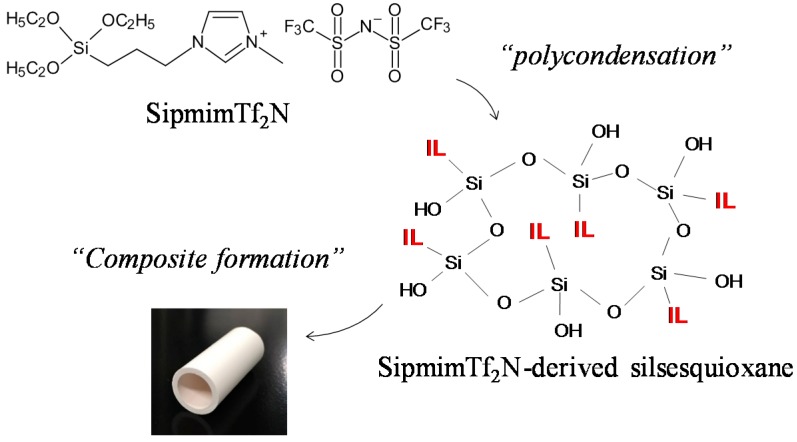
Concept of an ionic liquid organosilica (ILOS) membrane.

Here, we present a new concept of a chemically stabilized IL membrane, which is an organosilica membrane with IL-like properties, thus named an ionic liquid organo-silica (ILOS) membrane (Figure 1). In contrast to the previous stabilization method [23,24], polycondensed silylated ILs were introduced to the pores of substrates to form a composition. In this study, the toluene permeability and toluene/H_2_ selectivity of the ILOS membranes were investigated. Their permeation mechanism and stability were also compared with SILM.

## 2. Experimental

### 2.1. Preparation of Silylated Ionic Liquids

A silylated ionic liquid, 1-methyl-3-(1-triethoxysilylpropyl) chloride (SipmimCl), was synthesized from 1-methylimidazole (Tokyo Chemical Industry, Tokyo, Japan, >99%) and 3-chloropropyltrimethoxysilane (Tokyo Chemical Industry, >97%). A mixture of 1-methylimidazole and 3-chloropropyltrimethoxysilane was allowed to react at 343 K under stirring for 48 h. The reaction mixture was washed three times with diethyl ether (Nacalai tesque, extraction grade) and then dried under vacuum at 323 K. The structure of the resulting SipmimCl was confirmed by ^1^H NMR (JEOL, Tokyo, Japan, JNM ECS-400) and attenuated total reflectance infrared spectroscopy (ATR-IR, Shimazu, Kyoto, Japan, IRAffinity-1S). The ^1^H NMR results indicated that chemicals other than absorbed H_2_O were not contained in the product.

A 1-methyl-3-(1-triethoxysilylpropyl) imidazolium bis(trifluoromethylsulfonyl)imide (SipmimTf_2_N) was prepared from SipmimCl by the anion exchange method. A slight excess of equimolar potassium bis(trifluoromethylsulfonyl)imide (KTf_2_N, Kanto Chemical, Tokyo, Japan, >99.8%) was added to SipmimCl/acetonitrile solution to prepare SipmimTf_2_N, with KCl as a byproduct. The resulting slurry was stirred at room temperature for 120 h. The precipitate (KCl) was removed by filtration, and then the obtained solution was dried under vacuum to remove acetonitrile. For further purification, the obtained SipmimTf_2_N was dissolved in dichloromethane and an appropriate amount of water was added to remove unreacted SipmimCl as well as remaining KCl and KTf_2_N. This procedure was repeated 10 times. Dehydration treatment was conducted by adding an appropriate amount of magnesium sulfate, and the added magnesium sulfate was removed by filtration. Finally, the obtained solution was dried under vacuum to remove dichloromethane, and then purified SipmimTf_2_N was obtained. The structure of the resulting SipmimTf_2_N was confirmed by ATR-IR and mass spectrometry (JEOL, JMS-700). The result of mass spectrometry indicated that Cl anion could not be detected from purified SipmimTf_2_N.

### 2.2. Membrane Preparation

The ILOS membranes were prepared on a nanoporous SiO_2_/Al_2_O_3_ tube (purchased from eSep, Kyoto, Japan, tubes of 30 mm length, 10 mm inner diameter, 12 mm outer diameter). The tube consisted of a macroporous Al_2_O_3_ support, intermediate Al_2_O_3_ layers, and a nanoporous SiO_2_ layer with a pore size of 4 nm. A cross-sectional SEM image is shown in Figure S1. Synthesized SipmimTf_2_N (70 mmol) was dissolved in methanol (17.5 mL), and 5.25 mL of NH_3_ aqueous solution (1 mol/L) was added as a catalyst. The solution was stirred at 293 K for two days. After stirring, the solution was heated at 383 K to remove solvent and catalyst. Further drying was performed under vacuum for 24 h at 293 K. The tube was immersed in the resulting polycondensed SipmimTf_2_N, and heated at 363 K for 24 h. After heating, the excess solution was removed from the surface of the tube by tissue. Finally, the membrane was calcined at 453 K. The cross-sectional elemental mapping of the ILOS membrane was analyzed with an SEM (Hitachi, Tokyo, Japan, S-4800), equipped with an energy dispersive X-ray spectrometry (EDX) analyzer (Horiba, Kyoto, Japan).

To identify the structural change of SipmimTf_2_N, the residual SipmimTf_2_N liquid, after heating at 363 K, was also calcined and characterized by ATR-IR. A ZnSe crystal was used in the experiment and the resolution of the ATR-IR spectrometer was 1 cm^−1^.

For comparison, SILM was also prepared by impregnating the tube with as-made SipmimTf_2_N.

### 2.3. Toluene/H_2_ Separation Test

Separation of a binary mixture (1:3 (molar)) of toluene/H_2_ was conducted at 343 K. A schematic diagram of the separation test apparatus is shown in Figure S2. The binary mixture and N_2_, as a sweep gas, were fed into a feed and a permeate side of the membrane, respectively. The flow rates of H_2_ and N_2_ were controlled using a mass flow controller. Toluene was sent to the vaporizer by a syringe pump. The flow rates of H_2_, toluene vapor, and N_2_ were 50, 16.6, and 20 cm^3^/min, respectively. The total pressure on the feed side and the permeate side was kept at 0.12 and 0.1 MPa, respectively. The permeate stream was analyzed using a gas chromatograph (Shimadzu GC-8A). The toluene vapor and H_2_ permeation performance of the membrane was evaluated using permeance (mol m^−2^ s^−1^ Pa^−1^) and separation factor. The toluene/H_2_ separation factor was calculated as the ratio of toluene and H_2_ permeance. The detection limit of the toluene/H_2_ permeation test was 10^−12^ mol m^−2^ s^−1^ Pa^−1^.

To evaluate membrane’s durability, the membrane after the permeation test was dried under vacuum to remove absorbed toluene, and we calculated the amount of blow-out of IL by measuring the weight change before and after the permeation test.

## 3. Results and Discussion

### 3.1. ATR-IR Spectra of Silylated Ionic Liquids

**Figure 2:**
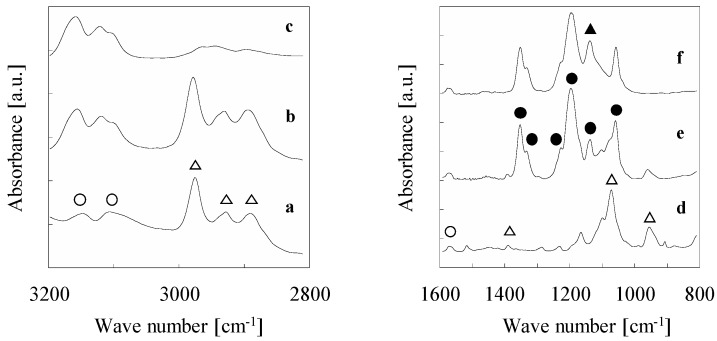
ATR-IR spectra of (**a**,**d**) SipmimCl; (**b**,**e**) SipmimTf_2_N; and (**c**,**f**) polycondensed-SipmimTf_2_N. (○ Imidazolium cation, △ -OC_2_H_5_ group, ● Tf_2_N anion, ▲ Si-O-Si).

The ATR-IR spectra of SipmimCl and SipmimTf_2_N before and after thermal treatment are given in Figure 2. In this study, we normalized the spectra using a peak around 1580 cm^−1^, which belongs to the imidazolium cation ring as a standard peak, and discussed the intensity of the peaks. The peaks in the spectral range of 1350–1000 cm^−1^, which belong to the S–N–S, S=O, and C–F stretching vibrations of the Tf_2_N anion [25], were not changed after the thermal treatment. The ATR-IR spectroscopy clearly shows that the anion exchange treatment was successfully conducted, and the structure of the Tf_2_N anion was not changed after the thermal treatment. Similar results were observed for the peaks in the spectral range of 3200–3050 cm^−1^. These peaks belong to the C–H stretching vibration of the imidazolium cation ring [26], indicating that the imidazolium cation ring also kept its structure after thermal treatment.

On the other hand, significant change was observed for the peaks in the spectral range of 3000–2850 cm^−1^ and at 960 cm^−1^. These peaks belong to the ethoxy group [27], and their peak intensity decreased after the thermal treatment. Additionally, the intensity of the Si–O–Si band (1130 cm^−1^) was increased [27], indicating that silica networks were formed through hydrolysis and condensation reaction of SipmimTf_2_N during the thermal treatment. Orel et al. studied structures of silylated IL, 1-methyl-3-(1-trimethoxysilylpropyl) imidazolium iodide, and its nanocomposite with tetramethoxysilane [27]. They studied the structure of the silylated IL in its non-hydrolyzed and hydrolyzed states, and in its fully condensed form by ATR-IR and ^29^Si NMR spectroscopic measurements, suggesting that ladder- and cube-like silsesquioxanes were formed as the most probable fully-condensed structure. Although further analysis is required for the structure of polycondensed SipmimTf_2_N in our study, similar ATR-IR spectra were obtained, indicating that ladder- and cube-like silsesquioxanes were formed after the thermal treatment.

### 3.2. Permeation and Separation Performance for H_2_/Toluene Mixture

**Figure 3:**
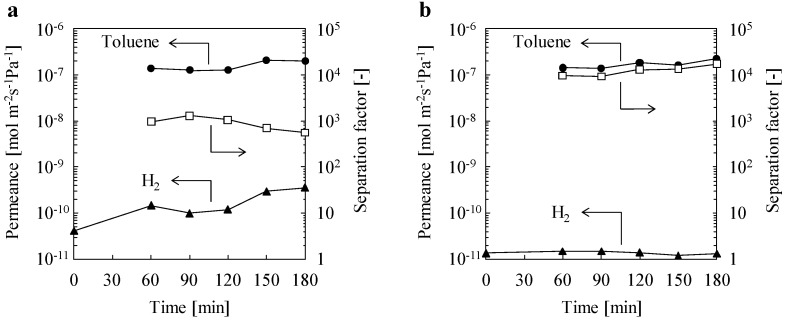
Time courses of toluene and H_2_ permeances and separation factor for (**a**) SILM and (**b**) the ILOS membrane. (● toluene permeance, ▲ H_2_ permeance, □ separation factor).

Figure 3 shows the time courses of toluene and H_2_ permeance and separation factors through the membranes. The separation tests ensure that the permeance values of toluene and H_2_ are accurate to two digits. The H_2_ permeances of 0 min were the values from a single H_2_ permeation test. The conditions of the single H_2_ permeation test were the same as those of the toluene/H_2_ separation test, except for the feed component.

The maximum toluene/H_2_ separation factor of SILM was 1280 after 90 min. The toluene and H_2_ permeances were 1.3 × 10^−7^ and 4.1 × 10^−11^ mol m^−2^ s^−1^ Pa^−1^, respectively (Figure 3a). The permeation mechanism of liquid membranes, including SILMs, is normally explained by the solution–diffusion mechanism. According to this mechanism, membrane selectivity is influenced by the membrane’s affinity to the feed molecules and the ease of diffusion of molecules through the membrane. Toluene can mix with SipmimTf_2_N. Thus, high permselectivity toward toluene of SILM is explained by the difference between the solubility of toluene and H_2_ in SipmimTf_2_N. Focusing on H_2_ permeance, the values for the binary mixture permeation test were larger than those for the single H_2_ permeation test, and increased with the permeation time. We also observed that the IL content in the tube after 180 min was decreased by 30% compared to that before the permeation tests. This blow-out of IL would affect the H_2_ diffusivity in SILM, resulting in the increase of H_2_ permeance.

The ILOS membrane showed a smaller H_2_ permeance than SILM for the single H_2_ gas permeation test (Figure 3b). After the addition of toluene in the feed stream, the H_2_ permeance did not changed significantly, and the membrane showed superior toluene permselectivity. The maximum toluene/H_2_ separation factor of the ILOS membrane was over 17,000 after 180 min. The toluene and H_2_ permeances were 2.3 × 10^−7^ and 1.3 × 10^−11^ mol m^−2^ s^−1^ Pa^−1^, respectively. The results of the permeation tests indicate that the permeation and separation mechanisms of the ILOS membrane are, as for SILMS, explained by the solution–diffusion mechanism. As opposed to chemically stabilized IL membranes [23,24], only polycondensed SipmimTf_2_N was used for preparation, and thus the pores of the tube were fully filled with polycondensed SipmimTf_2_N. Additionally, the silica network may be occupied by the branched IL, and hence H_2_ permeation pathways did not exist in the ILOS membrane. As for SILMs, permselectivity toward toluene is explained by differences in the solubility of toluene and H_2_ in polycondensed SipmimTf_2_N. Additionally, the viscosity of the SipmimTf_2_N after thermal treatment was increased from 37.6 mPa·s to >1000 mPa·s (over the detection limit) at 343 K, due to the formation of the silica network. The increased viscosity affected the diffusivity of H_2_ molecules in the ILOS membrane, leading to a decrease in H_2_ permeance compared to that of SILM. As a result, a toluene/H_2_ separation factor over 17,000 was obtained. The viscosity change also affected the toluene permeance, but significant differences could not be observed between SILM and the ILOS membrane. A possible reason for this is that the toluene permeation was not controlled by the diffusion of toluene through the membrane. In this permeation test condition, dissolution and/or desorption of toluene would be the rate-limiting step in both membranes. Compared to SILM, the amount of blow-out of IL from the tube was reduced to 3% after 180 min. Stabilization on the pore surface of the tubes and increased viscosity improved the durability of the membrane.

## 4. Conclusions

In this study, we demonstrated the advanced properties of an ILOS membrane with respect to toluene/H_2_ separation. The ILOS membrane showed a toluene/H_2_ separation factor greater than 17,000 based on a dissolution–diffusion mechanism with improved durability over SILM. This is the first report on the preparation of organosilica membranes using silylated IL as a precursor. The affinity of ILs to chemicals can be controlled by the combination of cation and anion. For example, amino-functionalized and basic ILs are able to capture CO_2_ based on chemisorption [20,22,28]. The ILOS membranes can be designed by using various IL structures to provide feasible and desirable separation performance for systems other than toluene/H_2_ as well, for example CO_2_/H_2_ and CO_2_/N_2_. Additionally, a cross-sectional EDX chemical map indicated that the IL existed not only in the nanoporous SiO_2_ layer, but also in a macroporous Al_2_O_3_ support (Figure S3). Thus, further improvements in the permeation performance of the ILOS membrane can be expected by decreasing its membrane thickness.

## Supplementary Materials

The following are available online at www.mdpi.com/1996-1944/10/8/901/s1, Figure S1: Cross-sectional SEM image of the tubular support, Figure S2: A schematic diagram of separation test apparatus, Figure S3: Cross-sectional (a) SEM image and (b) EDX chemical map for F atoms of the ILOS membrane. (White dot represents F atoms).
