# Supplementary material for: Organosilica Membrane with Ionic Liquid Properties for Separation of Toluene/H2 Mixture
Source: Materials (Basel). 2017 Aug 3;10(8):901. doi: 10.3390/ma10080901 (PMC5578267; doi:10.3390/ma10080901)
Supplement: Supplementary file 1 [file materials-10-00901-s001.pdf]

materials

Organosilica membrane with ionic liquid properties for separation of toluene/H_2 mixture

Yuichiro Hirota[a,*], Yohei Maeda[a], Yusuke Yamamoto[a], Manabu Miyamoto[b], and Norikazu Nishiyama

[a]Division of Chemical Engineering,

Graduate School of Engineering Science, Osaka University,

1–3 Machikaneyama, Toyonaka, Osaka 560–8531, Japan.

E-mail: yhirota@cheng.es.osaka-u.ac.jp

[b]Department of Chemistry and Biomolecular Science, Gifu University,

1-1 Yanagido, Gifu 501-1193, Japan.

Supplementary Materials

Table of contents

1. Cross-sectional SEM image of the tubular support
2. Toluene/H_2 separation test apparatus
3. Cross-sectional SEM image and EDX analysis of the ILOS membrane

1. Cross-sectional SEM image of the tubular support

Cross-sectional SEM image of the tubular support (purchased from eSep Inc.) is shown below.

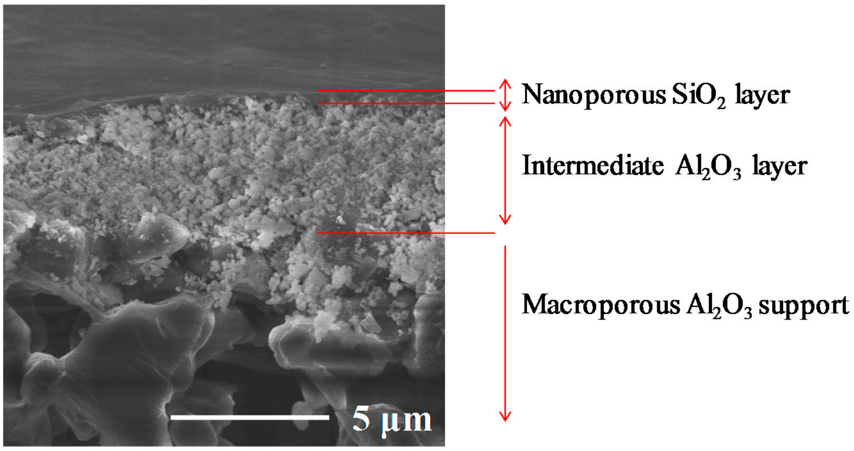

Figure S1. Cross-sectional SEM image of the tubular support.

2. Toluene/H_2 separation test apparatus

Separation of a binary mixture of toluene/H_2 (toluene : H_2 = 1 : 3 (molar)) was conducted at 343 K. A schematic diagram of separation test apparatus is shown in Figure S2. The binary mixture and N_2 as a sweep gas were fed into a feed and a permeate side of the membrane, respectively. Flow rates of H_2 and N_2 as a sweep gas were controlled using mass flow controller. Toluene was sent to vaporizer by a syringe pump. The flow rates of H_2, toluene vapor and N_2 were 50, 16.6 and 20 cm^3/min, respectively. The total pressure on the feed side and the permeate side was kept at 0.12 and 0.1 MPa, respectively. Permeate stream was analysed using a gas chromatograph (Shimadzu GC-8A). The toluene vapor and H_2 permeation performance of the membrane was evaluated based on permeance [mol $m^{-2}s^{-1}Pa^{-1}$] and separation factor. Toluene/H_2 separation factor was calculated as the ratio of toluene and H_2 permeance. The detection limit of toluene/H_2 permeation test was 10^{-12} mol $m^{-2}s^{-1}Pa^{-1}$.

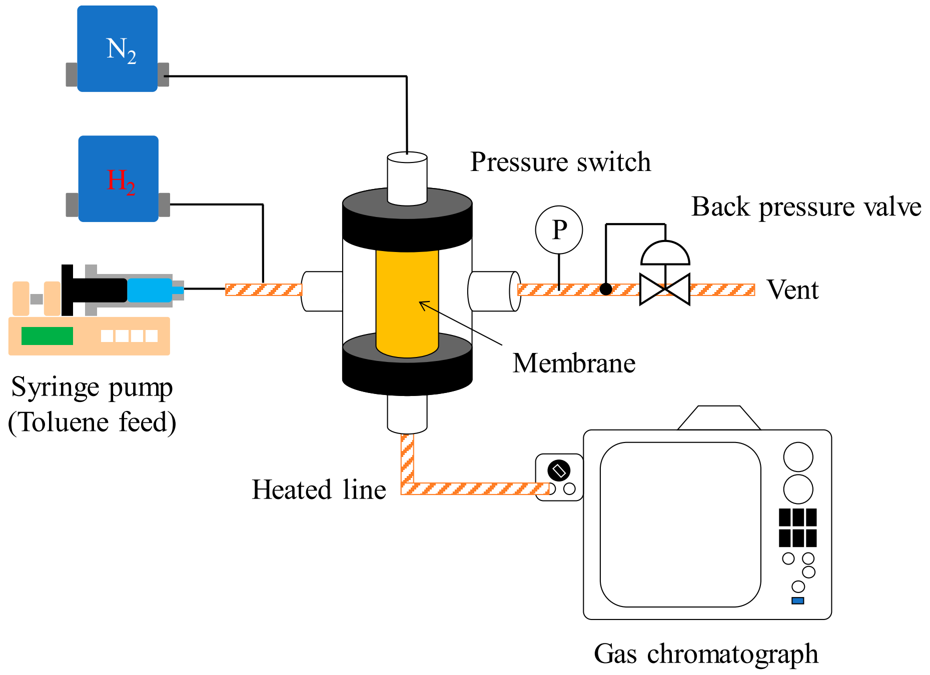

Figure S2. A schematic diagram of separation test apparatus.

3. Cross-sectional SEM image and EDX analysis of the ILOS membrane

Cross-sectional SEM image and EDX chemical map for F atom of the ILOS membrane were shown in Figure 3S. The IL was existed not only in nanoporous SiO_2 layer but also in a macroporous Al_2O_3 support.

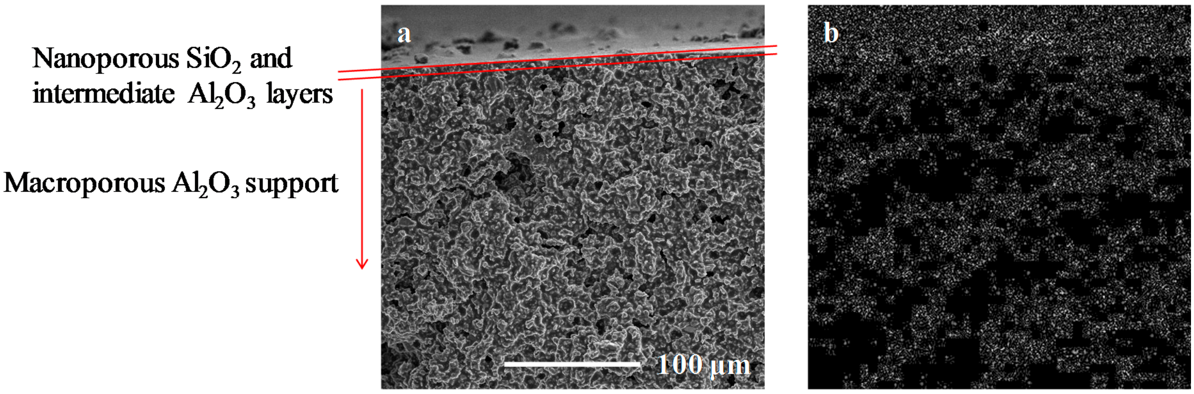

Figure S3. Cross-sectional (a) SEM image and (b) EDX chemical map for F atoms of the ILOS membrane. (White dot represents F atoms.)
